# Traumatic Experiences, Perceived Discrimination, and Psychological Distress Among Members of Various Socially Marginalized Groups

**DOI:** 10.3389/fpsyg.2019.00416

**Published:** 2019-02-28

**Authors:** Kimberly Matheson, Mindi D. Foster, Amy Bombay, Robyn J. McQuaid, Hymie Anisman

**Affiliations:** ^1^Department of Neuroscience, Carleton University, Ottawa, ON, Canada; ^2^The Royal Ottawa’s Institute of Mental Health Research, University of Ottawa, Ottawa, ON, Canada; ^3^Department of Psychology, Wilfrid Laurier University, Waterloo, ON, Canada; ^4^Department of Psychiatry and School of Nursing, Dalhousie University, Halifax, NS, Canada

**Keywords:** trauma, discrimination, appraisals, mental health symptoms, marginalized groups

## Abstract

Perceived discrimination has consistently been shown to be associated with diminished mental health, but the psychological processes underlying this link are less well understood. The present series of four studies assessed the role of a history traumatic events in generating a proliferation of discrimination stressors and threat appraisals, which in turn predict psychological distress (depressive and posttraumatic stress symptoms) (mediation model), or whether prior traumatic events sensitize group members, such that when they encounter discrimination, the link to stress-related symptoms is heightened (moderation model). Each of the studies assessed a different marginalized group in Canada, including Indigenous peoples, Blacks, Jews, and a diverse sample of women. Participants completed measures assessing history of traumatic events, perceived explicit and ambiguous discrimination, discrimination threat appraisals, and symptoms of depression and posttraumatic stress. The four populations varied in their experiences, with Indigenous peoples encountering the highest levels of trauma, discrimination, and psychological distress symptoms. A mediated model was evident among Indigenous peoples and women, possibly reflecting the role of systemic processes that engender discrimination when traumatic events are experienced. There was evidence for a moderating role of a history of traumatic events on the relations between discrimination and depressive symptoms among Jewish and Black participants. Although the hypothesized synergistic effects of traumatic experiences were noted when assessing the relation between perceived discrimination and depressive symptoms among Jews, the presence of trauma blunted these relations among Blacks. The results suggest that trauma-informed approaches to addressing stress-related processes and psychological outcomes need to consider the unique social context of members of various socially marginalized groups.

## Introduction

Compromised mental health has been associated with more frequent life stressors, and among socially marginalized^1^ populations experiences of individual and systemic discrimination may elicit particularly marked adverse consequences. Several reviews have concluded that discrimination has deleterious effects on a range of mental and physical health outcomes (Pascoe and Smart Richman, 2009; Williams and Mohammed, 2009; Pieterse et al., 2012; Schmitt et al., 2014; Paradies et al., 2015). The mechanisms for this relation remain unclear (McDonough and Walters, 2001; Williams and Mohammed, 2009; Lewis et al., 2015), although such outcomes may emanate from a combination of biological, environmental, social, and cultural factors that exacerbate or alleviate particular stress outcomes.

The implications of discriminatory encounters might vary in light of a group’s history of traumatic events (Ellis et al., 2008; Bombay et al., 2009; Priest et al., 2013; Sacks and Murphey, 2018). Such experiences might be more prevalent among socially marginalized groups as a result of lower socioeconomic status, the nature of the negative stereotypes and expectations applied to members of particular groups, normative roles that put them at greater risk, and/or because they have been subject to historical collective trauma that has intergenerational consequences (Bombay et al., 2009). Experiencing traumatic events may, in turn, be at the root of increased encounters with discrimination stressors (i.e., due to greater vulnerability or threat sensitivity to racism, sexism), and the proliferation of such discriminatory experiences (stress generation) may further compromise individuals’ mental health (Matheson et al., 2007; Liu and Alloy, 2010; Bombay et al., 2011). Alternatively, it is possible that trauma might not in itself render individuals more prone to encountering discrimination, but rather, when discriminatory experiences are superimposed on a backdrop of trauma, individuals may be more likely to suffer psychological distress. In effect, a history of traumatic events might overtax the coping resources the individual has available to contend with further stressors, thereby intensifying the stress reaction (Bombay et al., 2011, 2013). It was the goal of the present research to assess whether the relation between traumatic events and stress-related psychological outcomes (depressive or posttraumatic stress symptoms) emanates from a proliferation of discrimination stressors engendered by traumatic events (mediation model), or whether a history of traumatic events sensitizes group members so that later stressor encounters exacerbate negative psychological outcomes (moderation model).

The nature of discrimination experiences and their implications may vary across social groups (Carter, 2007; Thoits, 2010; Roberts et al., 2011), as well as among members of any given group (Lee, 2005; Thompson-Miller and Feagin, 2007; Pascoe and Smart Richman, 2009; Thoits, 2010; Bombay et al., 2013). Members of some groups may be especially likely to encounter discrimination because their membership in targeted groups is visible to others (e.g., based on skin color, language, etc.). Relationships to the dominant group also differ, for example, due to a capacity to ‘pass’ as a member of the dominant group, or because of social norms and roles that promote integration (e.g., gendered relationships). Thus, we assessed the patterns of relations among four groups that varied in the characteristics that shape their experiences of discrimination and that define the nature of their relationships to the dominant group, namely Indigenous peoples, Black and Jewish Canadians, and women.

### Traumatic Events and Discrimination Stressors Among Socially Marginalized Groups

Traumatic events may comprise those that are personal (e.g., abuse, unexpected loss of a loved one) or are collectively experienced (e.g., natural disaster, historical trauma). The relations with psychological distress likely depend on the availability of effective coping strategies (e.g., problem-solving, social support seeking), and processes for deriving meaning from the experience. For example, religious beliefs might enable individuals to find meaning in their traumatic experiences (God’s will) and thereby protect against the negative effects on mental health (Ysseldyk et al., 2011). Collective trauma can likewise be understood within a framework that focuses on survival and growth versus loss and victimization (Elsass, 2001; Abramowitz, 2005; Hatala et al., 2016).

In this regard, social roles, norms, and beliefs associated with ethnicity, gender, or religion have the capacity to alter the meaning of a traumatic event and its association with group membership (Barreto and Ellemers, 2005; Kubiak, 2005; Bryant-Davis, 2007; Carter, 2007). For instance, an experience of physical assault can be construed as race-based when the perpetrator targets the individual as a result of his/her ethnic group membership. The event may become further racialized as the victim contends with a justice system that differentially reacts to victims and perpetrators from different racial backgrounds, a health system that is differentially accessible to those of different racial backgrounds, and a child and family welfare system that differentially supports parents (and hence children) who struggle with discriminatory system biases. In effect, traumatic events that might not in and of themselves constitute an act of racism (or sexism) may result in the proliferation of events and experiences that are driven by race (or gender). The accumulation of these experiences can contribute to the evolution of psychological stress disorders, such as depression or posttraumatic stress disorder (PTSD) (Belle and Doucet, 2003; Kubiak, 2005; Pearlin et al., 2005). Indeed, persistent and protracted ‘micro’ or ‘insidious’ discrimination stressors (Pearlin et al., 2005; Sue et al., 2007) can elicit negative consequences equivalent to those provoked by more severe events (Kubiak, 2005; Anisman et al., 2008; Ellis et al., 2008; Rosen and Lilienfeld, 2008). Thus, for some groups, traumatic events might result in a proliferation of discrimination stressors that promote psychological distress.

It is also possible, however, that when discrimination is superimposed on a history of traumatic events, already stretched coping resources might be overwhelmed (allostatic overload), rendering individuals less able to ward off the threat to their well-being (McEwen, 2000). In this regard, traumatic events might evoke a sensitization of the neurobiological systems underlying stress disorders (Anisman et al., 2003). As a result, individuals may be more vigilant or reactive to subsequent stressor experiences of discrimination (Franklin and Boyd-Franklin, 2000). Such reactivity could be expressed through greater perceptions of having been the target of discrimination, or stronger appraisals that such events are a threat to well-being (i.e., an appraisal that one’s coping resources are insufficient to protect against negative outcomes). In this instance, rather than triggering the generation of further stressors, traumatic events might act synergistically with discrimination experiences and threat appraisals, rendering individuals more vulnerable to stress-related psychological symptoms (Cohen et al., 2007; Matheson et al., 2008).

The role of a history of traumatic events as either the triggering factor that generates discrimination experiences that undermine well-being (mediated relation), or sensitizes (moderates) individuals’ perceptions and appraisals of discrimination, might depend on the nature of the characteristics associated with a given marginalized group (Matheson et al., 2016). In particular, groups vary in terms of historical relationships with the dominant group, visibility and controllability of the identity (Quinn et al., 2014), as well as entrenched norms and belief systems that enable particular meaning-making frameworks (Barreto and Ellemers, 2005). While any number of disadvantaged social groups vary along these dimensions, in the proposed investigation four socially marginalized groups were considered, namely, Indigenous peoples, Blacks, Jews, and women in Canada. Although all of these groups are the target of prejudicial attitudes and stereotypes that diminish their social status, they have been differentially targeted by historical trauma, vary in the visibility of the features that define their group belonging and hence their ability to pass within the dominant group (with concurrent implications for the proliferation of stressors rooted in discrimination), and hold qualitatively different intergroup relationships based on social norms and roles. These features might contribute to differences in how group members react to traumatic events and discrimination. Analyses of the responses of such disparate groups facilitate the identification of both common and unique underlying processes by which trauma history and perceived discrimination are associated with vulnerability to psychological distress, particularly as members of each of these groups are known to be differentially at risk for stress-related disorders (Anisman et al., 2018). The hypotheses that we assessed within each group were whether:

(1) Traumatic events would be associated with a proliferation of discrimination stressors and threat appraisals, which in turn would be related to more severe depressive and posttraumatic stress symptoms (mediation model; stress generation).
(2) The relations between perceptions of discrimination and threat appraisals with depressive and posttraumatic stress symptoms would be especially pronounced when they occurred on a backdrop of a history of traumatic events (moderation model; stress sensitization).
(3) The role of a history of traumatic events in generating a proliferation of discrimination stressors or exacerbating the relations between discrimination perceptions/appraisals and psychological stress symptoms would vary across marginalized groups.

## Study 1

Indigenous peoples in Canada, and around the world, have been the target of colonialist practices and policies aimed at their subjugation, assimilation, and even eradication. In Canada, for the past two centuries, pervasive discriminatory policies that have been collectively referred to as cultural genocide (Truth and Reconciliation Commission [TRC], 2015), have resulted in the cumulative intergenerational impacts that constitute historical trauma (Brave Heart and De Bruyn, 1998; Bombay et al., 2014). One of the most egregious of these experiences in recent history was the Indian Residential Schools (IRSs), wherein several generations of Indigenous children were forcefully removed from their homes and communities, and were subjected to maltreatment and abuse by residential school staff. The intergenerational consequences of such trauma on the proliferation of stressors have been demonstrated. In particular, Indigenous adults with a parent who attended IRS reported greater exposure to adverse childhood experiences, which was associated with increased threat appraisals and perceptions of discrimination, and heightened depressive symptoms (Bombay et al., 2011, 2013).

It has been suggested that Indigenous peoples continue to be the most disadvantaged group in Canada, with instances of racism described as being ‘alarmingly high’ (Morrison et al., 2014; Statistics Canada, 2015). Indigenous communities and resources continue to be overseen by federal legislation (The Indian Act), and efforts to achieve equality and compensation for historical injustices, and their active exercise of treaty rights, are opposed by many non-Indigenous Canadians (Denis, 2015). In addition, they suffer disproportionate rates of physical illnesses (diabetes, HIV/AIDS, hepatitis, severe respiratory illnesses) and mental health disturbances (depression, posttraumatic stress, drug and alcohol addiction, suicide) (Kirmayer, 2014). Given the historical trauma, systemic biases, and the continued gaps that exist between Indigenous and non-Indigenous Canadians, it was expected that traumatic encounters would be especially likely to play a role in the proliferation of discrimination experiences, threat appraisals, and severity of stress-related psychological symptoms among Indigenous peoples.

### Methods

#### Participants and Procedure

Indigenous participants over the age of 18 years were recruited through advertisements posted at community/health centers across Canada and through various listservs. They were invited to participate in a survey either on-line or by having it mailed to them in paper format with a stamped addressed return envelope. Participants provided informed consent, completed the survey, received a gift certificate for their participation, and were debriefed. This study was approved by the research ethics board at Carleton University.

Participants (*N* = 354) self-identified as either First Nations (*n* = 257), Métis (*n* = 86) or Inuit (*n* = 11). Of those who were First Nations, only 29 reported living on-reserve. As seen in Table 1, participants were primarily female, and ranged in age from 16 to 76 years. Over a third of the sample had only a high school diploma or less, with the remainder having some form of post-secondary education. The majority were in a serious dating relationship, co-habitating or married. Almost two-thirds of the sample reported having at least one child.

**Table 1:** Demographic characteristics of the participants in each of the four studies.

	Study 1	Study 2	Study 3	Study 4
	Indigenous	Black	Jewish	Women
	(*N* = 354)	(*N* = 139)	(*N* = 212)	(*N* = 783)
**Gender**				
Male	87 (24.6%)	47 (33.8%)	73 (34.4%)	
Female	267 (75.4%)	91 (65.5%)	139 (65.6%)	783 (100%)
Age: Mean years (*SD*)	35.4 (11.5)	26.3 (9.8)	35.3 (16.2)	28.7 (11.6)
**Education**				
High school or less	129 (36.4%)	27 (19.5%)	17 (8.2%)	108 (14.1%)
Undergraduate/college	198 (55.9%)	106 (76.3%)	139 (67.5%)	574 (73.3%)
Postgrad/professional	27 (7.6%)	5 (3.6%)	50 (24.2%)	82 (10.5%)
**Relationship status**				
Single	85 (24.0%)	90 (64.7%)	74 (34.9%)	318 (40.8%)
Serious relationship	46 (13.0%)	26 (18.7%)	42 (19.9%)	210 (26.8%)
Married/cohabitating	198 (55.9%)	19 (13.7%)	85 (40.1%)	220 (28.1%)
Divorced/widowed	25 (7.0%)	4 (2.9%)	11 (5.2%)	31 (3.9%)
At least one child	230 (65.0%)	21 (15.1%)	86 (40.6%)	174 (22.2%)

#### Measures

The Traumatic Life Events Questionnaire (Kubany et al., 2000) is a self-report survey that assesses exposure to a broad spectrum of potentially traumatic events, ranging from natural disasters, accidents, assaults, and childhood abuses. We further included an item to assess whether the individual “had something happen to you that you believe represented an experience of discrimination (e.g., religious, racial, sex)?” Events were described in behaviorally descriptive terms. To distinguish between the perceived severity of traumatic events, we followed the DSM-IV guidelines for PTSD, in that respondents who indicated experiencing each event were further asked if they felt intense fear, helplessness, or horror at the time of the event. Although these trauma features are not incorporated in the DSM-5 as essential for the development of PTSD, their inclusion enabled us to distinguish the subjectively evaluated traumatic nature of the events experienced. This was especially relevant with respect to discrimination in order to distinguish it from day-to-day perceptions of discrimination.

Scores reflecting participants’ history of traumatic events were calculated by summing participants’ indication of whether they had experienced each of five types of traumatic events (based on Breslau et al., 1999), and also reported that at least one encounter of the event type had caused fear, helplessness, and/or horror. The event types included (1) the experience of a shocking or unexpected event (natural disaster, accident, living in a war zone, witnessing a violent event, experienced a life-threatening illness, miscarriage or abortion), (2) experiencing the unexpected death of someone close to them, (3) learning or seeing something negative happen to someone close to them who is still alive (accident, witnessing family violence when growing up), (4) assaultive experiences (childhood physical or sexual abuse, spousal assault, rape, stalked, or life threatened), and (5) discrimination.

The 13-item version of the Beck Depression Inventory (Beck and Beck, 1972) was used to assess severity of depressive symptoms. For each question, participants chose from one of four responses (coded 0 to 3), with each response reflecting increasing symptom severity. Summed scores were calculated, ranging from 0 to 39 (Cronbach’s α = 0.91). We previously found that the 13 and 21 item versions of the BDI yielded better than 95% agreement (Matheson et al., 2005).

Symptoms of posttraumatic stress were assessed using the 22-item Impact of Events Scale-Revised (IES-R) (Weiss and Marmar, 1997), which captures the three dimensions of traumatic stress, namely hypervigilance, perseverative, and intrusive thinking. Participants indicated how distressing each symptom had been for them in the past 7 days on a scale of 0 (not at all) to 4 (extremely), and responses were summed (Cronbach’s α = 0.96).

A modified 15-item version of the Perceived Ethnic Discrimination Questionnaire (Contrada et al., 2001) measured several types of explicit day-to-day discrimination toward Aboriginal (Indigenous) peoples, including verbal rejection (e.g., ethnic slurs, insults), avoidance (e.g., shunning), inequality-exclusion (e.g., denial of equal treatment or access), devaluation (e.g., actions expressing negative evaluations), and physical threat-aggression (e.g., actual or threatened harm). Participants indicated how often they had encountered each of these experiences in the past 12 months using a 7-point scale ranging from 1 (never happened) to 7 (happened very often). Responses were averaged across all items (Cronbach’s α = 0.96).

To assess threat appraisals regarding the experience of discrimination, participants considered “an event where you have been treated unfairly for being Aboriginal,” and appraised it in terms of ‘how threatening the event is in relation to how you feel about yourself,’ whether it ‘only affected you in a minor way, or do you feel that it affects almost everything you do,’ and ‘how stressed do you feel about this event?’ Responses were on 7-point scales, ranging from 1 (not at all) to 7 (extremely), with average scores reflecting high threat appraisal (Cronbach’s α = 0.78). Threat appraisals were moderately correlated with levels of perceived discrimination, *r* = 0.41, *p* < 0.001.

Finally, background variables (age, gender, education, relationship status, number of children) were assessed. In addition, questions that might pertain to participants’ exposure to systems and policies that affect the lives of Indigenous peoples were included, namely, parental and personal attendance at an IRS, being in foster care while growing up, and whether the participant lived on- or off-reserve.

#### Statistical Analyses

Mediation analyses were conducted to assess whether the number of traumatic events were associated with the proliferation of perceived discrimination and threat appraisals, and these in turn were linked to greater distress symptoms. Using Hayes (2017) macro, multiple mediation models (Model 4) were conducted to evaluate whether the indirect (mediated) effect of trauma events in relation to each of depressive and posttraumatic stress symptoms (separate analyses) through perceived discrimination and threat appraisals was significant. To assess whether number of traumatic events interacted with perceptions of discrimination and threat appraisals to predict psychological distress, moderation analyses were conducted using hierarchical regressions wherein depressive and posttraumatic stress symptoms were regressed onto the history of traumatic events, followed by perceived discrimination and threat appraisals, and lastly the interactions between number of trauma events and each of perceived discrimination and appraisals. Variables were mean centered prior to analysis. Where the moderating (interaction) effect of trauma events was significant, simple slope analyses at one standard deviation above and below the mean number of trauma events were conducted. Background variables [gender, age, education (ordinal scaling), relationship status (currently in a relationship or not), and whether they had children] that met the assumptions of analysis of covariance were included as covariates. Significant interaction effects between covariates and predictor variables were reported.

### Results and Discussion

#### Background and History of Traumatic Events

It has been suggested that Indigenous peoples in Canada are among the most discriminated against groups, with a pervasive history of colonization resulting in historical trauma. In line with this, among Indigenous participants, a history of traumatic events was common. A majority of Indigenous participants (90.1%) reported that they had experienced at least one type of traumatic event. As seen in Table 2, the most common forms of trauma experienced were physical or sexual assault, and witnessing the distress of a loved one. Based on additional background measures, 119 (33.6%) reported having at least one parent who attended IRS. As well, 67 (18.9%) participants indicated that they had been in foster care at some point while growing up, and 31 (8.8%) reported having attended IRS; all of these participants were among those who indicated experiencing at least one traumatic event. Although these child experiences were associated with greater rates of reporting all types of trauma, of those who had been in foster care, 91% indicated having witnessed something distressing to someone they cared about, and 91% reported that they had experienced some form of physical or sexual assault. Among those who attended IRS, 93.5% reported some form of traumatic assault and 87.1% had experienced the sudden death of a loved one. There were no significant differences on any of the variables of interest as a function of gender, relationship status, having children, or living on- or off-reserve (Table 3). Older participants were more likely to report a history of more types of traumatic events, *r* = 0.31, *p* < 0.001, and to perceive discrimination, *r* = 0.16, *p* = 0.005. Education was associated with less perceived discrimination, *r* = -0.15, *p* = 0.009, as well as less severe depressive, *r* = -0.14, *p* = 0.01, and posttraumatic stress symptoms, *r* = -0.14, *p* = 0.013.

**Table 2:** Number of participants (% of sample) experiencing each type of trauma in each of the four studies.

	Study 1	Study 2	Study 3	Study 4	χ^2^(*df* = 3)	
	Indigenous	Black	Jewish	Women	
	(*N* = 354)	(*N* = 139)	(*N* = 212)	(*N* = 783)	
At least one trauma	319 (90.1%)	89 (64.5%)	122 (57.5%)	623 (79.6%)	100.82^∗∗∗^
Shock	205 (57.9%)	40 (28.8%)	49 (23.1%)	233 (29.8%)	105.97^∗∗∗^
Death of loved one	225 (63.6%)	39 (28.1%)	51 (24.1%)	281 (35.9%)	117.01^∗∗∗^
Witness other distress	236 (66.7%)	57 (41.0%)	118 (55.7%)	485 (61.9%)	30.37^∗∗∗^
Assault	245 (69.2%)	31 (22.3%)	30 (14.2%)	207 (26.4%)	259.34^∗∗∗^
Discrimination	142 (40.1%)	37 (26.6%)	39 (18.4%)	126 (16.1%)	83.15^∗∗∗^

*^∗∗∗^*p* < 0.001.*

**Table 3:** Means (*se*) of predictor, mediating, and outcome variables in the four studies as a function of participant gender.

	Males	Females
**Study 1: Indigenous peoples**
Number of trauma events (0–5)	2.74 (0.18)	3.05 (0.09)
Depressive symptoms (0–39)	5.95 (0.65)	6.38 (0.37)
Posttraumatic stress symptoms (0–88)	30.83 (2.61)	30.06 (1.36)
Perceived (explicit) discrimination (1–7)	3.05 (0.18)	2.78 (0.09)
Threat appraisals (1–7)	3.21 (0.20)	2.99 (0.11)
**Study 2: Blacks**
Number of trauma events	1.28 (0.21)	1.60 (0.16)
Depressive symptoms	5.80 (0.84)	6.80 (0.68)
Posttraumatic stress symptoms	23.91 (2.89)	24.57 (2.33)
Explicit racial discrimination	2.88 (0.19)	2.43 (0.10)^∗^
Ambiguous racial discrimination	3.20 (0.20)	2.89 (0.13)
Threat appraisals (-5 to +5)	2.31 (0.26)	3.00 (0.18)^∗^
**Study 3: Jews**
Number of trauma events	0.89 (0.13)	1.42 (0.12)^∗∗^
Depressive symptoms	4.37 (0.74)	4.91 (0.44)
Posttraumatic stress symptoms	15.59 (2.42)	17.02 (1.54)
Explicit religious discrimination	1.75 (0.11)	1.57 (0.06)
Ambiguous religious discrimination	2.17 (0.11)	2.20 (0.08)
Threat appraisals (-5 to +5)	2.10 (0.22)	2.23 (0.14)
**Study 4: Women**
Number of trauma events	–	1.35 (0.04)
Depressive symptoms	–	5.49 (0.18)
Posttraumatic stress symptoms	–	21.09 (0.67)
Explicit gender discrimination	–	2.43 (0.03)
Ambiguous gender discrimination	–	2.83 (0.04)
Threat appraisals (-5 to +5)	–	2.07 (0.05)

*^∗^*p* < 0.05; ^∗∗^*p* < 0.01.*

#### Mediated Relations Between History of Traumatic Events and Psychological Distress

**FIGURE 1:**
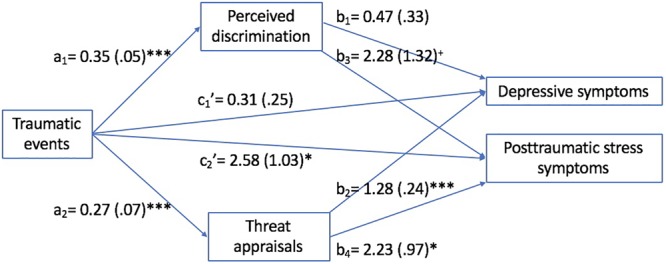
Unstandardized coefficients (standard errors) of models assessing mediating role of perceptions of discrimination and threat appraisals in the relation between number of types of traumatic experienced and distress symptoms (controlling education) among Indigenous peoples (Study 1). ^+^*p* < 0.10; ^∗^*p* < 0.05; and ^∗∗∗^*p* < 0.001.

Mediation analyses (with education included as a covariate) indicated, as seen in Figure 1, that a history of more traumatic events was associated with greater perceived discrimination and stronger threat appraisals; these mediators were positively correlated, *r* = 0.41, *p* < 0.001, and both were significantly correlated with depressive symptoms (*r*s = 0.40 and 0.43, *p*s < 0.001, respectively). When the mediators were included in the model, the relation between traumatic events and depressive symptoms was reduced to non-significance (confidence interval contained 0), c′ = 0.31 (*se* = 0.25), 95% CI [-0.18, 0.79], and the total indirect effect was significant, Effect = 0.51, 95% CI [0.20, 0.88], suggesting full mediation primarily as a result of heightened threat appraisals.

Posttraumatic stress symptoms (controlling education) were uniquely associated with greater perceived discrimination and greater threat appraisals (Figure 1). The direct relation between traumatic events and posttraumatic stress symptoms remained significant, c′ = 2.58 (*se* = 1.03), 95% CI [0.55, 4.60]. The total indirect mediation effect through both perceived discrimination and threat appraisals combined was also significant, Effect = 1.33, 95% CI [0.40, 2.45], suggesting a partial mediation model.

Thus, it would appear that encountering more traumatic events was accompanied by a proliferation of perceived discrimination stressors, which was associated with greater psychological distress. Although both perceived discrimination and threat appraisals were correlated with distress symptoms, the mediation was largely through greater threat appraisals. Such appraisals were likely confounded with the severity of the discriminatory encounters, and as a result played a more significant role in linking traumatic events with distress outcomes. This said, the threat appraisals reflected participants’ beliefs that their discrimination experiences were pervasive and profound, and so it is perhaps not surprising that these evaluations were more likely to account for stress-related psychological symptoms.

Although age was not linearly related to depressive symptoms, it was a significant moderator of the mediating role of threat appraisals, Moderated Mediation Index = -0.02, 95% CI [-0.04, -0.003]. The conditional indirect (mediated) effect of traumatic events on depressive symptoms through threat appraisals dissipated among participants who were older, Effect = 0.10, 95% CI [-0.17, 0.47], relative to those who were younger, Effect = 0.56, 95% CI [0.27, 0.91]. Thus, although older Indigenous participants reported more traumatic events and perceived greater discrimination, the mediated relations between traumatic events and depressive symptoms diminished among older participants. Perhaps with greater age, participants were better able to cope with such events. However, understanding the basis for this difference calls for a longitudinal perspective on trauma and experiences of discrimination, as it is likely that the events that are experienced and the meaning that is derived from them changes over time (Gee et al., 2012).

#### Moderating Role of Traumatic Events

Hierarchical regression analyses (controlling education) indicated that a history of traumatic events did not significantly moderate the relations between perceived discrimination and threat appraisals with depressive, *R^2^_cha_* = 0.012, *F*(2,201) = 1.56, *p* = 0.212, or posttraumatic stress symptoms, *R^2^_cha_* = 0.002, *F* < 1. The finding that a history of traumatic events did not exacerbate (moderate) the relations between perceived discrimination and threat appraisals in relation to symptomatology likely emanates from the extent to which the majority of Indigenous peoples experienced both historical and current traumatic and day-to-day negative encounters. Not only did a substantial number experience such stressors, the intergenerational effects of historical trauma might also prevail.

## Study 2

The Canadian Black community has existed for generations and has suffered from explicit social and institutional discrimination (Backhouse, 2001). However, the constitution of minority groups in Canada has shifted, in part due to increased immigration and asylum seeking from African countries over the past few decades (Statistics Canada, 2017), where many encountered collective trauma associated with civil war and ethnic genocide. Thus, there exists a history of traumatic events among this population, but to some degree, immigration to Canada allowed an escape from the collective trauma experience. Moreover, the explicit subjugation of Blacks is less ingrained in Canadian historical and continued legislative policies than is the case of Indigenous peoples. As a result, although their history of traumatic events is likely distressing, these experiences may be more likely to sensitize individuals to the negative effects of subsequent stressors, rather than eliciting a proliferation of further stressors. This said, Blacks are often highly identifiable in terms of their group membership vis-a-vis the White Euro-Caucasian majority, and while integration is certainly more common today than in previous generations, there continue to exist segregated ethnic urban areas (e.g., Ray and Preston, 2015). Study 2 assessed the relations among a history of traumatic events, discrimination perceptions and appraisals, and psychological distress among Black Canadians.

One of the challenges associated with measures of perceived discrimination is that underlying discriminatory intent is frequently ambiguous (“was that person’s reaction to me because of my group membership?”). Indeed, belonging to a marginalized group ought to be associated with identifiable experiences of discrimination, and members of such groups acknowledge that group-based discrimination exists, but at the same time they are often uncertain that they themselves have encountered discrimination (Crosby, 1984; Barreto and Ellemers, 2005). The uncertainty associated with determining whether one’s experience constitutes discrimination can itself be stressful, as the individual is concerned about whether the behavior of another was intentionally offensive, whether she or he merits such treatment, and whether his or her own behavior elicited such a reaction (Major et al., 2002; Quinn et al., 2014). As a result, it has been suggested that ambiguous discrimination encounters may elicit greater distress than explicit or blatant experiences, as individuals (in this instance, Black Americans) were better able to cope with explicit experiences (Bennett et al., 2004; Salvatore and Shelton, 2007). Thus, this study (and the two that follow with Jews and a diverse sample of women) further assessed perceptions of discriminatory experiences that were explicit as well as those that were more ambiguous in intent.

### Methods

#### Procedures

Participants for each of Studies 2–4 were recruited through advertisements posted on various websites, newspapers and community flyers, and signs posted on public bulletin boards of community centers, service organizations, and workplaces. They were invited to participate in a survey either on-line or by contacting us to mail out the survey in paper format, together with a stamped addressed return envelop. To retain diversity within each sample, and given the smaller numerical representation of ethnic (Black) and religious (Jewish) participant groups, 80% of females who identified as Black or Jewish were directed to Studies 2 and 3, respectively, whereas the remaining 20% were directed to Study 4, which focuses on women’s gendered experiences. Upon submission of their survey responses, participants were provided with a written debriefing and entered into an instant draw lottery for a $10 gift certificate, as well as a $500 lottery held at the end of data collection. These studies were approved by the research ethics boards at Carleton University and Wilfrid Laurier University.

#### Measures

On the whole, the materials used in Studies 2–4 were the same as those in Study 1, with some adaptations and additions. Reflecting the primary categories of traumatic events assessed in Study 1, participants reported on their experience of each of the five trauma types by indicating how often they recalled experiencing each event type, and whether, in the most serious instance, they experienced fear, helplessness or horror. An affirmative response to the latter indicated that the event had been subjectively experienced as traumatic. Total traumatic events scores were calculated by counting how many of the five event types had been encountered and experienced as traumatic, with total scores ranging from 0 to 5.

In addition to assessing explicit discrimination, 22 items were included to assess ambiguous aspects of discrimination, including concerns about stereotype confirmation (e.g., “concern that by *talking a certain way* you might appear to be confirming a stereotype about being Black”), and uncertainty regarding the experience (e.g., “Sometimes I’m not sure if what I’m seeing or hearing is racist”). Participants indicated how often they had each of these experiences using a 7-point scale ranging from 1 (never happened) to 7 (happened very often), and responses were averaged to reflect greater perceptions of ambiguous discrimination. In so doing, both perceived explicit (Cronbach’s αs across the studies ranged from 0.93 to 0.94), and ambiguous (αs ranged from 0.93 to 0.95) discrimination were assessed. Responses to the two forms of perceived discrimination were positively correlated (*r*s ranged from 0.53 to 0.67 across samples).

The threat appraisal measure was also changed in order to assess appraisals that were independent of the severity of participants’ own discrimination history. To this end, participants read a brief description of a series of eight hypothetical situations that varied in the extent to which a perpetrator’s negative behavior might be construed as targeting group membership (based on ethnic, religious, or gender background) (Foster, 2001). Using a rating scale ranging from -5 (not at all) to +5 (definitely/extremely), participants indicated whether they perceived each scenario to be ‘due to discrimination,’ ‘likely to be present again,’ ‘likely to occur in other situations,’ or would have ‘serious negative consequences personally.’ Average responses to these items across the eight scenarios constituted an index of threat appraisals (αs ranged from 0.89 to 0.90). Measured as such, threat appraisals were minimally to moderately correlated to explicit (*r*s ranged from -0.02 to 0.32) and ambiguous (*r*s ranged from 0.14 to 0.37) discrimination perceptions.

#### Participants in Study 2

Participants self-identified their ethnic background as Black (*N* = 139). The majority were female (Table 1), and ages ranged from 18 to 65 years. Most were Canadian citizens (79.9%) or landed immigrants (7.9%), although some were in Canada on temporary (student) visas (12.2%). The majority had some undergraduate or college education. Most reported their relationship status as single or casual dating. A minority of the sample reported having at least one child.

### Results and Discussion

#### Background and History of Traumatic Events

As seen in Table 2, almost two-thirds of Black participants experienced at least one type of traumatic event in their lifetime. The most common trauma experienced was witnessing the distress of a loved one, whereas rates of experiencing other forms of trauma were roughly equal, with just over a quarter of the sample experiencing each type. There were no gender differences in trauma types experienced. As seen in Table 3, there were gender differences in discrimination perceptions, in that, Black women were less likely to report encountering explicit discrimination based on ethnic background, even though they appraised the discrimination scenarios as more threatening. Likewise, younger participants were more likely to appraise situations of discrimination as threatening, *r* = -0.22, *p* = 0.037, whereas older participants were marginally more likely to report experiencing a greater number of trauma types, *r* = 0.15, *p* = 0.07. There were no significant differences associated with education, relationship status, or having children.

#### Mediated Relations Between History of Traumatic Events and Psychological Distress

To assess whether traumatic events rendered Blacks more likely to perceive explicit or ambiguous discrimination and to appraise discrimination scenarios as threatening, and whether these, in turn, contributed to depressive or posttraumatic stress symptoms, mediation models were tested. These models were not found to be significant, primarily due to the lack of significant relations between the number of traumatic events with either the mediator or outcome variables. Thus, rather than demonstrating a stress proliferation response associated with traumatic history, Black participants’ psychological distress in relation to traumatic or discrimination stressors was largely muted. This finding was unexpected, as there exist numerous studies, reviews, and meta-analyses indicating that discrimination is a stressful and traumatic experience that is predictive of poorer mental and physical health outcomes (Pascoe and Smart Richman, 2009; Pieterse et al., 2012; Paradies et al., 2015). This said, a systematic review that considered specific ethnic group memberships suggested that this relation was strongest among Asian and Latin American participants, compared to those who were African American (Paradies et al., 2015), and Black Canadians may well differ in this regard as well. Past findings associated with additional moderators of the relation between discrimination and indices of well-being have been inconsistent (Lewis et al., 2015).

#### Moderating Role of Traumatic Events

Hierarchical regression analyses predicting depressive symptoms indicated no significant main effects or interactions associated with number of traumatic events, *explicit* discrimination, or threat appraisals. However, as expected, the correlations indicated that perceptions of *ambiguous* discrimination were associated with more severe depressive symptoms, *r* = 0.23, *p* = 0.014.

**FIGURE 2:**
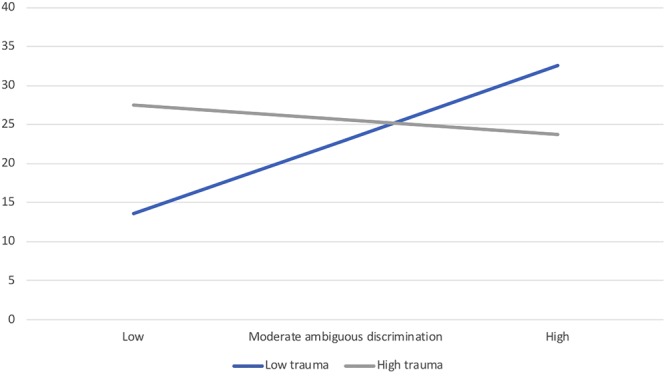
Moderating role of history of traumatic events on the relation between ambiguous discrimination perceptions and posttraumatic stress symptoms among Black Canadians (Study 2).

Posttraumatic stress symptoms among Black participants were marginally associated with perceived discrimination and appraisals, *R^2^_cha_* = 0.075, *F*(3,88) = 2.43, *p* = 0.071, reflecting only a unique relation between greater appraisals of threat and *lower* posttraumatic stress symptoms, β = -0.23, *p* = 0.028. The moderating role of traumatic events was significant, *R^2^_cha_* = 0.084, *F*(3,85) = 2.91, *p* = 0.039, particularly the interaction with ambiguous discrimination, β = -0.32, *p* = 0.013. As seen in Figure 2, it was in the *absence* of prior trauma (1 *SD* below the mean) that greater perceptions of ambiguous discrimination were associated with more severe posttraumatic stress symptoms, *b* = 7.60, *p* = 0.001; this relation dissipated in the presence of more frequent types of traumatic events, *b* = -1.47, *p* = 0.45. This finding runs contrary to our hypothesis that traumatic events would sensitize individuals, such that the relations between discrimination and distress symptoms were expected to be heightened in the presence of trauma history. Notably, as seen in Figure 2, posttraumatic stress symptoms appear to be relatively high among participants who encountered prior traumatic events. As a result, it might be in the absence of trauma that individuals had the cognitive and emotional resources to acknowledge ambiguous situations as potentially discriminatory, and in doing so, reported greater distress symptoms. Along similar lines, it has been found that, in contrast to reminders of other types of trauma (e.g., assault), when marginalized ethnic group members were exposed to reminders of discrimination, they demonstrated a blunted stress response (diminished cortisol reaction) (Matheson and Anisman, 2012). Although speculative, a blunted (rather than a sensitized) response might be the most adaptive reaction when coping resources are stretched, and the stressor is pervasive, unpredictable, and ongoing.

## Study 3

Antisemitism has existed since time immemorial, with one of the most recent violent genocidal efforts being the Holocaust. Considerable literature has revealed the role of intergenerational trauma on the psychological well-being of offspring of survivors of the Holocaust (Bowers and Yehuda, 2016), and this was linked to epigenetic alterations found in survivors and their offspring (Yehuda et al., 2016). This said, the transmission of trauma among Jewish offspring does not appear to be associated with perceptions of encountering more discrimination, and in particular, it was not so long ago that prominent Jewish North Americans argued that while “anti-Semitism is not gone,” it was “increasingly irrelevant to the daily lives and opportunities of Canadian Jews” (Weinfeld, 2001).

However, recent events in many countries have underscored that this optimistic view was largely incorrect, as antisemitism has progressively increased, including in Canada (B’Nai Brith, 2018). Indeed, the status of Jews has become more threatened than it has been in decades, most obviously seen in the pervasive anti-Israel actions that are disproportionate relative to attention paid to numerous domestic and global humanitarian crises. In this regard, many Jews believe that the escalation of antisemitism is often couched in terms of anti-Zionism (which not all Jews view as a challenge to their group identity) (B’Nai Brith, 2018). This confounding of political and racial attitudes creates ambiguity as to whether the individual or even the group has been a target of discrimination. Given the historical trauma of this population, individuals’ history of traumatic events might render them more sensitive to discriminatory sentiments when they are encountered, resulting in greater stress-related psychological symptoms.

North American Jews are highly assimilated, such that many pass within the dominant group virtually invisible. In this regard, having the choice of whether to reveal group membership may further alter the vigilance of individuals, and influence anticipation and sensitivity to antisemitic discrimination experiences. To the extent that such heightened vigilance occurs, as in other devalued groups, Jews may be at risk for psychological distress.

### Methods

#### Participants and Procedure

Participants (*N* = 212) self-identified as Jewish and, as seen in Table 1, were primarily female, and ranging in age from 17 to 81 years. Most were Canadian citizens (96.7%) or landed immigrants (2.8%). The majority had some undergraduate or college education or higher post-graduate or professional degrees. Many reported their relationship status as either single or were co-habitating/married. Under half reported having at least one child.

As it was of interest to characterize the historical trauma encountered by Jewish participants, included in the history of traumatic events measure was an item asking whether ‘either of your parents were directly part of the Holocaust.’

### Results and Discussion

#### Background and History of Traumatic Events

Over half of Jewish participants reported that they had experienced at least one type of traumatic event. As with Indigenous and Black participants, the most common type of trauma encountered was witnessing the distress of a loved one (Table 2). Rates of reporting other trauma types were roughly equal, with under a quarter experiencing each type. In addition, 19 participants indicated that they had a parent who had survived the Holocaust; all but six of these were among those who indicated experiencing trauma as a result of witnessing the distress of a loved one. The only gender difference was the number of trauma types experienced, in that women reported more types of traumatic events than did men (Table 3). Examination of each of the trauma types indicated that only assault showed a gender difference, χ^2^(1) = 9.24, *p* = 0.002; of the 30 Jewish participants who reported traumatic assault, 27 were female. Older Jewish participants reported more types of traumatic events, *r* = 0.21, *p* < 0.003, but were less likely to perceive ambiguous instances of discrimination, *r* = -0.15, *p* = 0.037. Level of education was also associated with less severe depressive symptoms, *r* = -0.20, *p* = 0.016, and marginally fewer symptoms of posttraumatic stress, *r* = -0.14, *p* = 0.082.

#### Mediated Relations Between Number of Trauma Events and Psychological Distress

Mediation analyses (with education included as a covariate) indicated that reports of more traumatic events were associated with greater perceived explicit, *a_1_* = 0.10 (*se* = 0.042), *p* = 0.021, and ambiguous discrimination, *a_2_* = 0.15 (*se* = 0.060), *p* = 0.013, but not higher threat appraisals, *a_3_* = 0.13 (*se* = 0.094), *p* = 0.158. In turn, although taken together these variables significantly predicted depressive symptoms, *R^2^*= 0.122, *F*(5,141) = 3.94, *p* = 0.002, none accounted for unique variance. Neither the total, *c* = 0.331 (*se* = 0.30), 95% CI [-0.26, 0.93], nor direct effect, *c*′ = 0.089 (*se* = 0.30), 95% CI [-0.51, 0.68], of traumatic events in relation to depressive symptoms was significant, but the total indirect mediated effect was, Effect = 0.24, 95% CI [0.03, 0.60]. The lack of unique relations suggests redundancy in the predictive utility of the indicators of perceived explicit and ambiguous discrimination, particularly given the significance of their bivariate correlations with depressive symptoms (*r*s = 0.24 and 0.28, *p*s < 0.01, respectively), and their correlation with each other, *r* = 0.60, *p* = 0.001. Neither the direct nor mediated relations between traumatic events and posttraumatic stress symptoms was significant, although both explicit, *r* = 0.14, *p* = 0.044, and ambiguous discrimination, *r* = 0.23, *p* = 0.003, were correlated with greater symptoms. In effect, among Jewish participants, traumatic events only indirectly influenced stress symptoms to the extent that such events generated a proliferation of discrimination stressors.

#### Moderating Role of Traumatic Events

**FIGURE 3:**
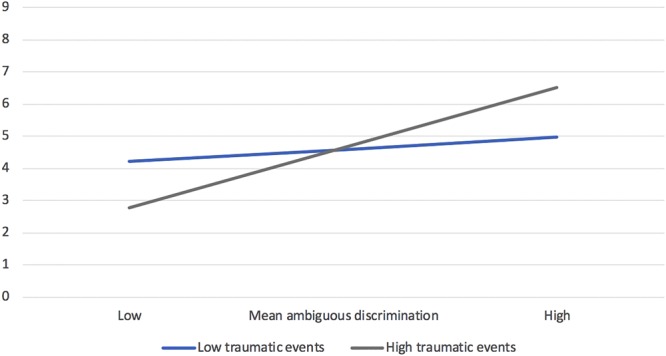
Moderating role of history of traumatic events on the relation between ambiguous discrimination perceptions and depressive symptoms (controlling education) among Jewish Canadians (Study 3).

Hierarchical regression analyses predicting depressive symptoms (controlling education) indicated that history of traumatic events was a significant moderator of the relation between perceived ambiguous discrimination and depressive symptoms, β = 0.27, *p* = 0.016. As hypothesized, and shown in Figure 3, as the number of types of traumatic events experienced increased, the relation between perceptions of ambiguous discrimination and depressive symptoms strengthened. In essence, among Jewish participants, it seems that traumatic events primarily served to sensitize individuals to the negative effects of discrimination experiences, particularly those involving uncertainty. This said, among Jewish participants, a history of traumatic experiences was not a significant moderator of the relations between discrimination perceptions and appraisals with posttraumatic stress symptoms.

Recall that older Jewish participants reported more traumatic events and perceived fewer instances of ambiguous discrimination. Age was also found to moderate the relation between explicit discrimination perceptions and depressive symptoms, β = -0.30, *p* = 0.004. Explicit discrimination was associated with greater depressive symptoms among younger participants, *b* = 3.01 (*se* = 0.87), *p* < 0.001, but this relationship diminished with age, *b* = 0.99 (*se* = 0.69), *p* = 0.155 (at 1 *SD* above the mean age). The reason for this is not entirely clear, and it could be that for older participants the nature of the explicit discrimination experiences paled relative to the discrimination trauma that had been encountered historically. As with the Indigenous sample in Study 1, this finding calls for taking a closer look at the longitudinal or age-cohort implications of discrimination for the well-being (Gee et al., 2012).

## Study 4

Although it is a common belief that women are no longer at a disadvantage, objective indicators reveal that women in Canada continue to experience inequities in social, occupational, and educational settings (Racco, 2017). Women are visible members of their group, but they are unique in the extent to which their lives are intertwined with the dominant group, men, in that, on an everyday basis, women interact in partnership with men, including husbands, brothers, fathers, co-workers, and so on. As a result, there exists a strong motivational basis to maximize intergroup harmony, and to minimize the extent to which behaviors that maintain women’s disadvantaged status are regarded as discriminatory (Kaiser, 2007; Garcia et al., 2010; Matheson et al., 2012). Indeed, even as gender roles are changing, there are multiple social ideologies within Western society (e.g., notions of a meritocracy) that, arguably, serve to maintain gender role segregation, and mask the discriminatory basis for women’s continued disadvantaged status (Crosby, 2004; Crosby et al., 2004). Such ideologies promote an interpretation of women’s experiences as individually based and personally controllable (Crosby, 2004). Thus, women’s experiences of discrimination are often ambiguous in terms of whether they emanate from personal, rather than group-based attributes (Barreto and Ellemers, 2005; Foster, 2009). In Study 4, it was expected that entrenched gender norms and expectations may result in a cascade of discriminatory experiences derived from having to contend with traumatic events. Given that such norms promote personal attributions for stressor challenges, discrimination events were expected to be associated with greater stress-related symptoms.

### Methods

#### Participants and Procedure

Participants were 783 women ranging in age from 16 to 85 years. Almost all were Canadian citizens (94.4%). The majority of those who identified their ethnic background were Euro-Caucasian (*n* = 607, 77.5%), with the largest ethnic minority group self-reporting as Asian (*n* = 95, 12.1%). Participants also self-identified as South or East Asian (*n* = 23, 2.9%), Black (*n* = 14, 1.8%), Indigenous (*n* = 12, 1.5%), Hispanic (*n* = 10, 1.3%), or Middle Eastern (*n* = 9, 1.1%); 13 participants identified themselves as other (unspecified or mixed ethnicity) or did not respond. As seen in Table 1, most women had some undergraduate or college education. Many reported their relationship status as single or casual dating. Less than a quarter of the sample had at least one child.

### Results and Discussion

#### Background and History of Traumatic Events

Almost 80% of women had experienced at least one type of traumatic event in her lifetime. As in the previous studies, the most common trauma experienced was witnessing the distress of a loved one (Table 2). In addition, over a third had experienced the unexpected death of a loved one, and over a quarter had experienced assault.

There were variations in trauma experiences as a function of women’s ethnic group membership. Indigenous (100%) and Black women (85.7%) were more likely to indicate at least one type of traumatic experience. Specifically, Indigenous (75.0%) and Black (50.0%) women were more likely to report unexpected loss due to death, χ^2^(6) = 20.09, *p* = 0.003, compared to other ethnic groups of women. They were also more likely to witness the distress of a loved one (Indigenous, 75.0%; Black, 64.3%), particularly relative to Asian/South-East Asian women (42.1%/47.8%), χ^2^(6) = 20.30, *p* = 0.002. Indigenous women (41.7%) were most likely to report a discrimination-related traumatic event, χ^2^(6) = 19.35, *p* = 0.004, and assault (58.3%), χ^2^(6) = 12.23, *p* = 0.057. There were no differences as a function of ethnic group membership in reports of experiencing an event involving traumatic shock, *p* = 0.26.

Older women reported experiencing more types of traumatic events, *r* = 0.14, *p* < 0.001, and were less likely to perceive encountering explicit, *r* = -0.13, *p* < 0.001, and ambiguous discrimination, *r* = -0.20, *p* < 0.001. Education was not related to history of traumatic events or perceptions of explicit discrimination, but was related to more perceived ambiguous discrimination, *r* = 0.08, *p* = 0.039, and greater threat appraisals, *r* = 0.08, *p* = 0.023, as well as less severe depressive, *r* = -0.12, *p =* 0.001, and posttraumatic stress symptoms, *r* = -0.11, *p* = 0.002. Number of children was not related to psychological distress outcomes, but was associated with more traumatic events, *r* = 0.20, *p* < 0.001, and less perceived ambiguous discrimination, *r* = -0.15, *p* < 0.001.

#### Mediated Relations Between History of Traumatic Events and Psychological Distress

Mediation analyses (controlling education) indicated that encountering more traumatic events was associated with greater perceived explicit, *a_1_* = 0.19 (*se* = 0.026), *p* < 0.001, and ambiguous discrimination, *a_2_* = 0.13 (*se* = 0.036), *p* = 0.001, as well as with stronger threat appraisals, *a_3_* = 0.18 (*se* = 0.043), *p* < 0.001. Of these mediators, only perceptions of explicit, *b_1_* = 0.96 (*se* = 0.28), *p* = 0.001, and ambiguous discrimination *b_2_* = 0.45 (*se* = 0.20), *p* = 0.029, uniquely predicted depressive symptoms. With these mediating variables included in the model, the direct effect of traumatic events in relation to depressive symptoms was reduced to non-significance c′ = 0.28 (*se* = 0.15), 95% CI [-0.017, 0.58]; the total indirect effect was significant, Effect = 0.28, 95% CI [0.16, 0.42], through both explicit, Effect = 0.18, 95% CI [0.07, 0.31], and ambiguous discrimination, Effect = 0.06, 95% CI [0.001, 0.13].

The mediation model predicting symptoms of posttraumatic stress (controlling education) demonstrated both a significant direct and indirect effect of traumatic events. Both perceptions of explicit, *b_1_* = 3.32 (*se* = 0.98), *p* = 0.001, and ambiguous discrimination *b_2_* = 3.25 (*se* = 0.72), *p* < 0.001, predicted posttraumatic stress symptoms. In this instance, the direct effect of traumatic events remained significant, c′ = 3.06 (*se* = 0.54), 95% CI [2.01, 4.12], as were the indirect effects through both explicit, Effect = 0.62, 95% CI [0.23, 1.07], and ambiguous discrimination, Effect = 0.40, 95% CI [0.13, 0.74].

Given systemic gender biases within social and organizational structures, together with social norms that might promote women attributing such events to personal characteristics, evidence of a stress proliferation model accounting for women’s stress-related psychological symptoms is not surprising. Perhaps emanating from these same social norms and expectations, the negative implications of explicit gender discrimination were more evident in relation to women’s distress than was seen in the previous samples. This might be a methodological issue, given the equivalent correlations between the two forms of discrimination with both traumatic events and posttraumatic stress symptoms, rendering their joint inclusion redundant in the mediation model. It might also be that such explicit encounters of discrimination were experienced as more jarring for women, particularly given the nature of their integrated relationship with men. In fact, given social norms that discourage claims of discrimination, it might well be that women’s perceptions of discrimination emanated from the emotional distress caused by particular interactions, rather than the reverse. However, if this was the case, one might have expected threat appraisals to have played a greater role in the mediation pathways.

#### Moderating Role of Traumatic Events

A moderation analysis (controlling education) predicting depressive symptoms indicated that there was no significant moderating role of history of traumatic events. Similarly, traumatic events did not moderate the relations between perceived discrimination or threat appraisals and posttraumatic stress symptoms.

## Comparative Analyses Across the Four Studies

It was expected that variations in the processes linking traumatic events, perceived discrimination and threat appraisals with stress-related psychological symptoms may exist as a result of the differences in the trauma contexts of marginalized social groups, and their subsequent experiences of discrimination. To evaluate whether such differences existed, common measures across the four studies were statistically compared. Significant differences for nominal variables were interpreted in relation to adjusted residuals of observed versus expected frequencies; continuous variables were followed up using Tukey’s adjustment for pairwise comparisons (α = 0.05).

The participants in the four studies differed in terms of background characteristics. As seen in Table 1, Indigenous and Jewish participants were older than the Blacks and Study 4 women, *F*(3,1390) = 46.7, *p* < 0.001, and they reported having more children, *F*(3,1390) = 156.6, *p* < 0.001. In addition, Indigenous peoples were more likely to have only up to a high school education, whereas Jewish participants were most likely to have completed a postgraduate degree, χ^2^(6) = 133.3, *p* < 0.001. Indigenous peoples were also more likely to be married or cohabitating with a partner, while Black participants were more likely to report being single, χ^2^(9) = 152.3, *p* < 0.001.

As noted across the studies, some types of traumatic events were more commonly experienced than others, and in particular, witnessing something negative happen to someone close who is still alive (accident, witnessing family violence when growing up) was common. In addition, as seen in Table 2, the groups differed in the extent to which they encountered all forms of trauma. Specifically, Indigenous participants were most likely to encounter traumatic events of every type. The sample of women in Study 4 were also more likely than Blacks or Jews to report at least one traumatic event, and in particular, both the sudden or unexpected death of a loved one and assault. Finally, like Indigenous participants, Blacks were more likely than Jews or women (in Study 4) to report traumatic discrimination encounters.

The four study samples also differed in terms of perceived explicit discrimination, η^2^= 0.118, *F*(3,1290) = 57.50, *p* < 0.001, symptoms of depression, η^2^= 0.015, *F*(3,1290) = 6.35, *p* < 0.001, and posttraumatic stress symptoms, η^2^= 0.048, *F*(3,1290) = 21.62, *p* < 0.001. Follow-up comparisons indicated that, as seen in Table 3, Indigenous participants perceived the most explicit discrimination, and reported the most severe depressive and posttraumatic stress symptoms, whereas Jewish participants were least likely to report discrimination or distress symptoms. Black participants and the diverse sample of women in Study 4 fell in between, although Blacks were not significantly different from Indigenous participants in reported depressive symptoms.

## General Discussion

A primary objective of the present study was to assess whether a history of traumatic events was associated with a proliferation of perceived discrimination stressors, which in turn would be associated with stress-related psychological symptoms (mediation model), or whether traumatic events sensitized group members to experience stronger distress reactions when discrimination was perceived (moderation). As expected, the role played by a history of traumatic events was found to vary across the groups considered.

A pattern of mediation was most consistently evident, emerging among Indigenous peoples and women, and to some degree among Jews. In these three groups, encountering more types of traumatic events was accompanied by greater perceptions of discrimination, and among Indigenous peoples and women, stronger threat appraisals were also reported. Moreover, consistent with past research demonstrating the negative implications of perceived discrimination for mental and physical health (Williams and Mohammed, 2009; Pascoe and Smart Richman, 2009; Schmitt et al., 2014; Paradies et al., 2015), perceptions of discrimination were consistently related to both greater depressive and posttraumatic stress symptoms. Perceptions of events that were explicit versus ambiguous in terms of whether they constituted discrimination were examined separately in Studies 2 through 4. Reports of these types of discrimination experiences were correlated with one another, and hence appeared redundant in some of the analytical approaches. In particular, among Black and Jewish participants, consideration of ambiguous situations accounted for additional variance in psychological distress, whereas perceptions of explicit discrimination did not. In effect, the uncertainty associated with such events may result in greater vigilance, as well as questioning of whether or not the experience is one in which the individual is being treated negatively due to personal inadequacies, which ultimately may elicit a stronger stress response (Carter, 2007; Williams and Mohammed, 2009). Perhaps for this reason, among women in Study 4, both ambiguous and explicit discrimination predicted stress-related psychological symptoms, as it was suggested that social roles that guide gender interactions should ordinarily dictate against such blatant negative interactions.

Less consistent across the four studies were the patterns supporting the possibility that traumatic events would moderate the relations between perceptions and appraisals of discrimination with psychological distress. The hypothesized exacerbation of distress when individuals encounter more traumatic events was found in the relation between perceptions of ambiguous discrimination and depressive symptoms among Jews. Among these group members, greater discrimination was associated with more severe depressive symptoms when it occurred on a backdrop of a history of traumatic events. In this instance, it might be that, in the absence of the strong social norms and policies that pertain to women and Indigenous peoples, among Jews, traumatic experiences are unlikely to trigger a cascade of experiences that might be rooted in systemic discrimination. Instead, the combination of traumatic experiences and the vigilance elicited by situational ambiguity (given their level of social integration and capacity to pass within the dominant group) served to promote the wear and tear on emotional, cognitive, and biological systems, thereby rendering these individuals more prone to stress-related disorders, such as depression (McEwen, 2000).

Among Black participants, a different pattern of moderation emerged that was not predicted. Specifically, there was a relation between perceptions of ambiguous discrimination and more severe depressive symptoms, but only when trauma experiences were minimal or absent. Despite the fact that there was ethnic diversity among the women in Study 4, the sample sizes associated with different ethnic minority groups were not sufficient to do comparative analyses to determine whether the distinctive results among the Black participants in Study 2 were replicated. This said, there is a growing body of research regarding the intersectionality of identities, demonstrating that women of different ethnic backgrounds respond differently to their experiences of gender discrimination. For example, in a national survey, among White women, problem-oriented coping with discrimination was *negatively* linked to avoidant coping strategies (e.g., emotional withdrawal), whereas among ethnic minority women, a problem-solving orientation (including social support seeking) was *positively* associated with avoidant strategies (Matheson and Anisman, 2012). It was almost as though avoidant coping among minority women reflected a collective response consciously employed to diminish the potential impacts of discrimination, a strategy that might be at play among the Black participants in Study 2. Other research has suggested that when discrimination was perceived as pervasive, ethnic minority women were more likely to cope through acceptance than active problem-solving (Matheson and Foster, 2013), and encountering sexist, rather than racist events, was more strongly associated with lower well-being among ethnic minority women (Settles, 2006; Remedios et al., 2012). In addition, Black women who attribute an event to racism, or the combination of racism and sexism, reported greater stress and reduced self-esteem (King, 2003). Thus, it appears that the effects of discrimination associated with multiple group identities are not straightforward.

Although one of the goals of the present study was to determine whether there were differences in the relations among traumatic experiences, perceived discrimination, and psychological distress across socially marginalized groups, explanations for the variations of patterns of findings across the four studies are *post hoc* and require empirical validation and longitudinal analyses to determine causal directions. The groups assessed varied inherently in terms of the features that might contribute to differences in how they react to trauma (based on collective historical, intergenerational, and personal experiences), and discrimination. The groups varied in the visibility of the features that define their belonging, enabling some to pass, and in the extent to which their segregation or integration with the dominant group is entrenched in legislative policies or prevailing social norms. In addition, the background data and comparative analyses indicated that the groups differed in terms of the severity of events experienced, with Indigenous peoples encountering considerable personal trauma and discrimination. Indigenous peoples also differed on other characteristics, including the likelihood of being in a personal (supportive) relationship with a partner, having children, and levels of education. It should therefore not be surprising that the resources available and strategies for coping with stressors and the resulting implications for well-being would also vary across groups. The increasing attention being paid to such factors in clinical contexts intended to support members of marginalized groups who present with psychological symptoms appears well placed (Bryant-Davis, 2007; Kirmayer and Craffa, 2014; Comas-Díaz, 2016).

Participants in the present study were largely urban (and off-reserve), and they needed to be comfortable with completing quantitative surveys of this form, creating a self-selection bias in our samples. Even in the studies of the different cultural groups, women were more strongly represented than men. These factors may limit the generalizability of the present findings with respect to the populations considered. There was a substantial age range in each of the studies, which allowed for differences among age cohorts to emerge. Indeed, these analyses suggested that among Black and Jewish participants, the relations between discrimination perceptions and psychological distress dissipated in the older cohorts. Others have called for a developmental perspective (and preferably longitudinal, as opposed to the correlational cohort design of the present series of studies) to understand the implications of the age at which traumatic events and discrimination experiences occur, their effects on physical and mental health, and the processes by which some individuals are rendered more resilient or vulnerable to poorer outcomes (Gee et al., 2012; Lewis et al., 2015).

The present study also relied on individuals’ subjective perceptions of whether discrimination had occurred. Such an approach holds merit, in that, the subjective experience of stressors is a primary process underlying the evolution of stress-related symptoms (Anisman, 2014). In this regard, whether the situation did or did not, in fact, constitute discrimination is almost irrelevant – indeed, there are psychological biases that lead individuals to either minimize objective evidence of discrimination, or to be hypervigilant to cues that might constitute discrimination (Kaiser and Major, 2006; Lewis et al., 2015). In this regard, the present studies differentiated between aspects of such perceptions, including whether events were perceived as blatant discrimination behavior versus situations in which they were uncertain whether the event reflected discrimination. In addition, although the measure of threat appraisals in Study 1 (Indigenous peoples) primarily assessed perceptions of the pervasiveness and impact of the individual’s own discrimination encounters, the threat appraisal measure employed in the remaining studies attempted to untie this evaluation from participants’ own experiences by asking them to consider a series of hypothetical situations that varied in the extent to which discrimination cues were explicit or ambiguous. In effect, this latter appraisal measure tapped into individuals’ vigilance, together with their concerns about the pervasiveness and threat associated with particular patterns of group-based treatment. The current findings indicated that the relations between discrimination perceptions and distress prevailed when such appraisals were controlled.

## Conclusion

In conclusion, the present series of studies contributes to a growing body of work demonstrating the negative associations between perceived discrimination and psychological symptoms, and further demonstrated the consistency between these relations across different marginalized populations in Canada. As organizations move toward policies of cultural safety, as well as programs and practices that are trauma-informed, there is a pressing need to understand the nature of the links between the traumatic experiences and discrimination among members of socially marginalized groups, including the specificities associated with each of the groups. While a sensitization model has played a dominant role in understanding how prior traumatic experiences can exacerbate reactions to subsequent stressor exposures, such that sometimes relatively innocuous stressors appear to trigger psychological disorders such as depression or PTSD (Anisman et al., 2003, 2018), such interactive effects were not consistently evident across the groups examined in the present study. Alongside a call for personalized treatments for mental illnesses, consideration of cultural and social contexts in which individuals are embedded may be critical to the success of such approaches. Indeed, without such contextual considerations, treatment approaches risk contributing to the proliferation of stressors socially marginalized group members must contend with following traumatic events (Kirmayer and Craffa, 2014; Matheson et al., 2018).

## Author Contributions

KM took the lead on analyses and write-up. All authors contributed to the conception of the studies, the interpretation of the results, and added intellectual content to the write-up. All authors have approved the content for publication, and are accountable for the accuracy or the reported results.

## Conflict of Interest Statement

The authors declare that the research was conducted in the absence of any commercial or financial relationships that could be construed as a potential conflict of interest.

## Footnotes

1 Numerous terms can be used to refer to social groups that are the target of personal, group, and systemic discrimination, including ‘marginalized,’ ‘stigmatized,’ ‘minority,’ or ‘disadvantaged.’ Each has a slightly different meaning with respect to the salient characteristics of the group, and intergroup processes and outcomes. Given the variation among groups that the present study considered, we have chosen to use the term ‘marginalized’ to reflect each group’s lower social status relative to the dominant group, and that membership in the group constitutes a basis for experiencing negative stereotypes, attitudes, and discrimination.
